# Assessment of multidrug-resistant *Listeria monocytogenes* in milk and milk product and One Health perspective

**DOI:** 10.1371/journal.pone.0270993

**Published:** 2022-07-06

**Authors:** Adeoye John Kayode, Anthony Ifeanyi Okoh

**Affiliations:** 1 SAMRC Microbial Water Quality Monitoring Center, University of Fort Hare, Alice, South Africa; 2 Applied and Environmental Microbiology Research Group, Department of Biochemistry and Microbiology, University of Fort Hare, Alice, South Africa; 3 Department of Environmental Health Sciences, University of Sharjah, Sharjah, United Arab Emirates; Universita degli Studi della Campania Luigi Vanvitelli, ITALY

## Abstract

The occurrence and the antibiogram signatures of *Listeria monocytogenes* (Lm) recovered from 65 milk samples and its products within the Eastern Cape province were examined. The EN ISO 11290:2017 procedures Parts 1 and 2 described by the International Organization for Standardization for the enumeration and isolation of Lm was adopted for the study. Lm was detected in 18.46% of all the samples examined, and the strains recovered from the samples belong to serotypes 4b and 1/2b. The virulence determinants including *prf*A, *plc*A, *plc*B, *inl*A, *inl*C, *hly*, *mpl*, *act*A, *inl*J and *inl*B were detected in all the isolates. About 95.24% of the studied Lm isolates demonstrated potential capacity for biofilm formation. The antibiogram profile revealed high resistance against sulfamethoxazole (71.43%), trimethoprim (52.86%); erythromycin, cefotetan and oxytetracycline (42.86% respectively). About 85.71% exhibited multiple antibiotic resistance phenotypes against the test antibiotics. The resistance determinants encoding resistance against the β-lactamase antibiotics [such as the *bla*_TEM_, *bla*_SHV_, *bla*_TEM_ *variants* (TEM-1 and TEM-2) and the *bla*_Z_], the tetracycline resistance genes (including *tet*A, *tet*D, *tet*G and *tet*M and *tetK*) were detected among resistant isolates. In addition, the aminoglycoside resistance gene *aph (3)-IIa (aphA2)*^*a*^ was detected only in one isolate. Finally, the sulfonamide resistance genes including the *sul*2 and the *sul*1 genes were the most frequently observed among Lm isolates. Generally, 71.43% of all Lm isolates recovered from the samples investigated harboured one or more resistance genes encoding resistance against various antibiotics. The antibiogram signatures of Lm isolates observed in this study is an indication that empirical treatment of listeriosis may be challenging in the future as the pathogen may obliterate the success of antibiotics. We, therefore, advocate for the recognition of the One Health approach to ensuring food safety and curbing the spread of antimicrobial resistance in food.

## Introduction

Milk is an essential human dietary requirement, constituting a significant percentage of the most widely consumed protein [1]. It is often classified among the essential sources of nutrients for humans due to its excellent nutritional composition, including minerals, vitamins, and protein [1]. However, its safety for human consumption is a concern in the food sector despite the increasing demand, especially for raw milk. For instance, there is a perception among consumers that heat treatment could destroy the nutritional and health benefits of raw milk [1, 2]. Notwithstanding, heat treatment has a beneficial bactericidal effect against contaminating microbes. Most of the contamination problems encountered in the dairy industry could be related to the minimal/unhygienic practices during processing although, post pasteurization contamination may occur from the plant environment [1, 3, 4]. Raw milk, together with other milk-based products, has been involved in several outbreaks in the past decades, probably because it offers an excellent medium for the growth of spoilage and pathogenic microbes including, *Listeria monocytogenes* (Lm) [5–7]. More worrisome, lactating cows could shed Lm in milk for a long time as a consequence of mastitis [8, 9]. As such, Lm could be unavoidably present in raw milk.

Lm is a Gram-positive, facultatively anaerobic bacteria of the family Listeriaceae widely distributed and abundant in nature. It is the etiological agent responsible for animal and human listeriosis [10]. Lm is most times known for sporadic outbreaks normally characterized by high case fatalities ranging from 20–30%. Symptoms including, gastroenteritis, headache, myalgia, pneumonia, meningitis, septicemia, fetal loss/abortion often manifest and the severity of the symptoms may be dependent on the state of health of an individual. More severe symptoms may be observed in infants, pregnant women, elderly persons, particularly those with comorbidities [10, 11]. The virulence traits (*act*A, *hly*, *iap*, *plc*B, *plc*A, *prf*A and *mpl*) regulated by the *Listeria* Pathogenic Islands (LIPI-1) and the internalin genes (*inl*A, *inl*B *inl*C *and inl*J) located on the LIPI-2 practically contribute to the severity of the infection [11, 12]. Lm is resilient and can persistently colonize food processing environments. It has the ability for biofilm formation; a unique trait that enhances its proliferation in harsh environments [10, 13]. Also, the microscopic size often makes its presence go unnoticed in food. These characteristics put together suggest why it is a serious threat to the food chain [10].

Lm has been reported in several foodborne outbreaks resulting in severe health consequences and economic losses [14, 15]. The first human listeriosis outbreak involving 142 cases associated with Mexican-style cheese claimed 48 lives including 18 adults, 10 neonates and 20 fetuses in 1985 in California [16]. Switzerland also went through two episodes of listeriosis outbreaks traced to locally made soft cheese in 2005 involving 10 patients, 5 deaths including 3 elderly and 2 abortions [17]. Unpasteurized milk (raw milk) from Pennsylvania dairy was mentioned in a multi-state listeriosis outbreak involving 2 individuals from California and Florida. Both patients > 65 years old were hospitalized in 2014 and the latter died [6].

In spite of the health significance of listeriosis infection, the development of resistance against valuable therapeutic agents could further complicate human listeriosis [18]. The occurrence of antimicrobial resistance and the acquisition of antibiotic resistance genes (ARGs) in Lm could become a complex public health emergency, especially in the food sector. Several reports about resistant Lm strains recovered from milk and milk products have been documented [18–21]. More worrisome is the increasing reports of multidrug-resistant (MDR) isolates after the first MDR isolate was identified in 1985 [18, 22]. The acquisition of genetic elements has immensely contributed to the increased prevalence of resistant Lm isolates [23]. Also, the indiscriminate use of sanitisers in processing plants and exposures to food processing stress could facilitate the development of resistance against clinically relevant antibiotics [23].

The economic and dietary significance of milk products cannot be overemphasized in many countries in Africa as it constitutes the most widely consumed animal protein [24]. Considering the nutritional significance of milk and milk products, the high demand for raw milk and the potential health risks associated with consumption of contaminated products, hence the need for monitoring to ensure its safety for human consumption. We, therefore, investigated the prevalence, the virulence signatures and the antibiogram profile of Lm recovered from dairy samples in the Eastern Cape Province, South Africa (ECPSA).

## Materials and methods

### Study location

This study was carried out in the Amathole, Chris Hani and Sarah Baartman Municipality Districts (MD) all located within the ECPSA—the second largest province in South Africa. Amathole MD is centrally located within the province, Chris Hani MD is situated in the North-Eastern region while Sarah Baartman MD is situated in the Western part of the ECPSA. The MDs are majorly made up of agrarian communities with notable commercial activities including agro-processing in the region which could be due to their proximity to ports in East London and Port Elizabeth. Samples were collected from nineteen sampling locations (towns/cities) located within the MDs. Amathole DM sampling locations include 1, 2, 3, 6, 8–11, 13 and 14; Chris Hani sampling locations include 12, 15 to 18; Baartman—4, 5, 7 and 19 (S1 Table in S1 File).

### Sample collection and presumptive counts of *L*. *monocytogenes* (Lm)

**Fig 1:**
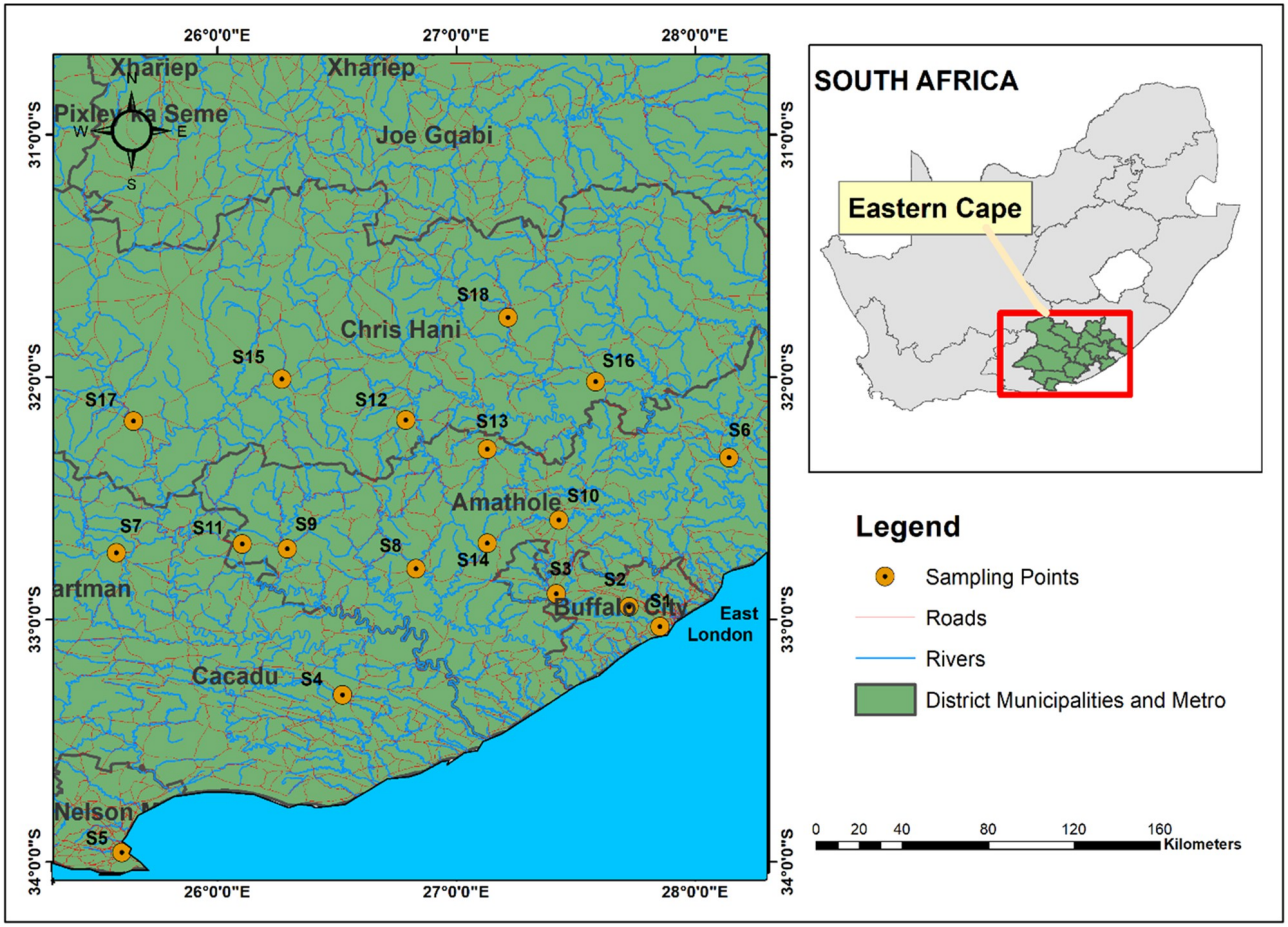
Map illustrating the sampling points within the Amathole, Chris Hani and Sarah Baartman District Municipalities, Eastern Cape Province, South Africa.

Raw milk samples (n = 26) from bulk tanks were collected in 1-litre sterile bottles. Pasteurized milk/fresh milk (n = 25) and cheese (n = 14) were collected from retail stores in sterile plastic bags. Sampling was done between February and September 2019 at the Amathole, Chris Hani and Sarah Baartman District Municipalities in the ECPSA (Fig 1). Ethical clearance (no: OKO041SKAY01) was obtained from the University of Fort Hare ethics committee before the study commenced. Samples were labelled and conveyed to the laboratory for analyses in an insulated ice packed container. The ISO (International Organization for Standardization) EN ISO 11290–2:2017 was adopted for the enumeration of presumptive Lm counts [25]. Twenty-five (25 ml) of raw milk and fresh milk samples were dispensed into 225 ml of buffered peptone water (CM1049 Oxoid Ltd, UK) and were serially diluted in ten folds replicates. For cheese samples, 25 g were weighed and aseptically stomached in 225 ml of peptone water (CM1049 Oxoid Ltd, UK) and were serially diluted in ten folds replicates. A 0.5 ml of appropriate dilutions were spread using a spiral platter on Chromogenic *Listeria* Agar (ISO) Base (CM1084 Oxoid Ltd, UK) supplemented with (ISO OCLA) differential supplement (SR0244E Oxoid Ltd, UK) and selective supplements (SR0226E Oxoid Ltd, UK) and Brilliance *Listeria* Agar Base (CM1080 Oxoid Ltd, UK) supplemented with Brilliance differential supplement (SR0228E Oxoid Ltd, UK) and selective supplements (SR0227E Oxoid Ltd, UK). Incubation was performed at 37°C between 24–48 ± 2h in aerobic conditions. Typical representative colonies obtained were counted and expressed CFU/g or CFU/ml (colony-forming units per gram).

### Isolation of presumptive *L*. *monocytogenes* (Lm)

The method of the International Organization for Standardization (EN ISO 11290–1:2017) for Lm was adopted in this study [25, 26]. Twenty-five (25 ml) of raw milk and fresh milk samples were taken while 25 g of cheese were aseptically stomached for pre-enrichment. The samples were pre-enriched in 225 ml of Half-Fraser Broth Base (CM0895) supplemented with half Frazer selective supplement (SR0166E Oxoid Ltd, UK) and incubated at 30°C for 24 h to resuscitate the pathogen. After pre-enrichment, 0.1 ml of the broth was added to a 10 ml Fraser broth for secondary enrichment in the selective medium and incubated for 24 to 48 h at 37°C). The broths were surface-plated on Chromogenic *Listeria* Agar (ISO) Base (CM1084 Oxoid Ltd, UK) supplemented with OCLA (ISO) differential supplement (SR0244E Oxoid Ltd, UK) and selective supplement (SR0226E Oxoid Ltd, UK), and Brilliance *Listeria* Agar Base (CM1080 Oxoid Ltd, UK) supplemented with Brilliance differential supplement (SR0228E Oxoid Ltd, UK) and Brilliance selective supplement (SR0227E Oxoid Ltd, UK). The plates were incubated aerobically at 37°C for 24–48 h. Each sample was analysed in triplicate and representative distinct colonies obtained were subcultured on nutrient agar for purity. Pure cultures of presumptive isolates were preserved on glycerol stock at -80°C.

### Extraction of DNA template

DNA extraction was done using the direct boiling method [27]. The DNA was quantified to determine its concentration in a fluorometer (Invitrogen Qubit fluorometer, Turner BioSystems).

### Identification and Serotyping of *L*. *monocytogenes* (Lm) isolates

Identification of *Listeria monocytogenes* isolates using specific primer sets *iap*-F: ACAAGCTGCACCTGTTGCAG and *iap*-R: TGACAGCGTGTGTAGTAGCA that targets *iap* (invasion associated protein) gene for Lm was done [28]. Isolates identities were further confirmed by amplicon sequencing. Multiplex PCR technique previously documented [29] was adopted for the molecular classification of Lm to various serotypes. The five primer sets used for this assay were mixed at final concentrations of *prs* 0.2 M; 1M for ORF2819, ORF2110, Imo0737, and 1.5M for Imo1118. Referenced strains of Lm (ATCC 19118 and ATCC 7644) were used as a positive control.

### Detection of virulence determinants

Virulence determinants (*inl*A, *inl*B, *inl*C, *inl*J, *act*A, *prf*A, *hly*A, *plc*A, *plc*B and *mpl*) genes of the Lm isolates were amplified using the primer sets (S2 Table in S1 File) reported previously by Jung et al. and Du et al. [30, 31]. The PCR products generated were separated by loading 5 μl amplified DNA products in 1.5% agarose gel stained with ethidium bromide resolved at 100 volts for 45 min suspended in 5 × TBE buffer using an agarose gel electrophoresis system (ADVANCE Mupid™-One-Takara, Japan) and detected with Alliance 4.7 UV trans-illuminator (Alliance XD-79.WL/26MX, France).

### Evaluation of biofilm-forming potential

The potential of biofilm-forming ability was evaluated using the microtiter plate biofilm production assay [32, 33]. Fresh overnight cultures were centrifuged (7,000 rpm for 2 min) and the cell pellets were washed and suspended in sterile buffered saline (PBS, pH 7.2) and adjusted to 0.5 McFarland [32]. About 20 μl of the previously standardized cell suspensions were inoculated in triplicates into sterile 96-well polystyrene microtiter plates containing 180 μl of sterile Tryptone Soy Broth (TSB) [33]. The microtiter plates were covered with lids and incubated at 37°C for 72 h. The TSB served as negative controls while Lm strains (ATCC 19118 MediMark ^®^ Europe and ATCC 7644 Mast diagnostics group Ltd, Merseyside U.K.) served as the positive control. After incubation, the contents of the plates were aspirated and washed in PBS (200 μl) 3 times to remove unattached cells. About 200 μl of 98% ethanol was dispensed to fix cells attached to the wells and allowed to air dry. The fixed cells were stained with 2% crystal violet (200 μl) for 30 min and excess stains were removed in distilled water and air-dried. About 200 μl of 35% acetic acid was added to the wells to resolubilize the crystal violet on a shaker (Orbit^™^ 1900 High-Capacity Lab Shaker, Labnet International, Inc., United States) for 30 min before the absorbance was read at 595 nm (OD595 nm) by a microtiter photometer (Synergy™Mx Monochromator-Based MultiMode Reader w/Time-resolved fluorescence, BioTek Instruments, United States) [34]. The optical density (OD595 nm) recorded for all positive and negative controls (OD_NC_) were computed to obtain the mean and standard deviation. The results obtained were used to categorized Lm isolates as either negative = (OD≤OD_NC_), weak = (OD_NC_ < OD595 nm≤2 × OD_NC_), moderate = (2 × OD_NC_ < OD595 nm ≤ 4 × OD_NC_) or strong = (4 × OD_NC_ < OD595 nm) biofilm formers [35].

### Profiling for antibiogram susceptibility

The disc diffusion method (Kirby Bauer) of the standard procedure was described by the Clinical and Laboratory Standards Institute [36] and the European Committee on Antimicrobial Susceptibility Testing [37]. The isolates were tested against a panel of 22 antibiotic discs (Mast Diagnostics, Oxoid, UK) for the treatment of microbial infections (S3a Table in S1 File). A 100 μl fresh culture of each bacterial cell suspension was transferred into a sterile 0.89% saline solution adjusted to 0.5 Mc Farland standard and spread plated on prepared Mueller-Hinton agar plates. Antibiotic discs were dispensed on the surfaces of the inoculated plates and the plates were incubated at 37°C for 24 hours. The zones of inhibitions were measured to the nearest millimetres (mm) after incubation. Classification of isolates as resistant (R), intermediate (I) or susceptible (S) to a particular antibiotic was based on the result obtained using standard reference values according to [36, 37] documents.

### Antibiotic resistance phenotypes/index (M)ARPs and (M)ARI

The multiple/antibiotic resistance patterns (MARPs) of Lm in respect of the antibiotics tested were applied to each isolate that shows phenotypic resistance against three or more antibiotics and indexed for MARI scores [38]. The MARI was computed thus:

$$\text{MARI}=\frac{no.\;of\;antibiotics\;to\;which\;isolate\;was\;resistant}{no.\;of\;antibiotics\;to\;which\;isolate\;was\;exposed}$$ (1)

Furthermore, the Antibiotic Resistance Index (ARI) was calculated for each of the dairy samples as described by Krumperman [38]. Thus, ARI for the samples was computed.


$$\text{ARI}=\frac{\text{aggregate}\;\text{antibiotic}\;\text{resistance}\;\text{score}\;\text{of}\;\text{all}\;\text{isolates}\;\text{from}\;\text{sample}}{\text{no}.\;\text{of}\;\text{antibiotics}\;\times \;\text{no}\;\;\text{of}\;\text{isolates}\;\text{from}\;\text{the}\;\text{sample}}$$ (2)

The multiple antibiotic resistance patterns, the frequency of resistance, the total sum of antibiotics to which the isolates exhibited resistance and isolate resistance against two or more classes of antibiotics (multidrug-resistant, MDR) were described.

### Screening for antimicrobial resistance genes among *L*. *monocytogenes* isolates

Genetic determinants of various antibiotic resistance (44) that encode the expression of tetracycline (*tet*A, B, C, D, E, G, K, L and M), chloramphenicol (*cat*I, *cat*II and *cml*A1), sulphonamides (*sul*1 and *sul*2), aminoglycosides [*str*A, *aad*A, *aac (3)-IIa (aacC2)*^*a*^, *aph (3)-Ia(aphA1)*^*a*^, *aph(3)-IIa (aphA2)*^*a*^] resistance were screened by simplex/multiplex PCR techniques. The sequences of primers, PCR protocols, and amplicon sizes are as described in our previous report [39]. Also, antibiotic resistance genes (ARGs) that encode *amp*C β-lactamases and extended-spectrum of β-lactamases variants (ESBLs), and carbapenems and the *bla*_TEM_ and *bla*z genes were screened for using simplex and multiplex PCR techniques as described elsewhere [40]. The PCR products generated were separated by loading 5 μl amplified DNA products in 1.5% agarose gel (Merck, SA) stained with ethidium bromide (Sigma-Aldrich, USA) resolved at 100 volts for 45 min suspended in 5 × TBE buffer using an agarose gel electrophoresis system (ADVANCE Mupid™-One-Takara, Japan) and detected with Alliance 4.7 UV trans-illuminator (Alliance XD-79.WL/26MX, France).

### Statistical analysis

Data obtained were subjected to statistical analysis to compared Lm counts and biofilm formation using one-way analysis of variance (ANOVA). The statistical significance of mean ± SD was considered at (p ≤ 0.05). The correlation in the distribution of phenotypic and genotypic resistance genes was done using Spearman’s correlation. Significant differences were identified at (p ≤ 0.01) and (p ≤ 0.05) as appropriate.

## Results

### Prevalence and detection of *L*. *monocytogenes* (Lm)

Sixty-five dairy samples including fresh milk from the bulk tank, pasteurized milk and cheese collected from groceries/retail stores at different geographical locations within the three municipalities districts. The presumptive Lm counts ranged between 2.0 × 10^3^ CFU/ml to 2.6 × 10^5^ CFU/ml for fresh milk samples, 2.0 × 10^3^ CFU/ml to 2.0 × 10^5^ CFU/ml fresh milk samples, and 2.0 × 10^3^ CFU/g to 1.6 × 10^4^ CFU/g for cheese samples. One-way analysis of variance (ANOVA) revealed dairy products significantly (p **<** 0.05) influence the mean counts (mean ± SD).

Twenty-one (n = 21, 13.55%) Lm were confirmed from 155 presumptive Lm isolates recovered from dairy samples (S1a and S1b Fig in S1 Raw images). The highest prevalence was observed in cheese (n = 12, 57.14%), while 7 (33.33%) was observed in fresh milk and 2 (9.52%) was observed for raw milk. Lm was detected in 12 (18.46%) of all the dairy samples collected. Six (42.86%) samples from cheese 4 (16%) of fresh milk samples and 2 (7.69%) of raw milk samples tested positive for Lm among the dairy samples analyzed (Table 1).

**Table 1:** Occurrence, serotypes, phenotypic and genotypic determinants of *L*. *monocytogenes* (Lm) virulence recovered from dairy samples.

Sample type	Positive dairy (%)	Prevalence of confirmed Lm (%)	Distribution (Lm) serotypes in dairy samples (%)		Biofilm formation (%)			
			1/2b	4b	Negative	Weak	Moderate	Strong
Cheese	6/14 (42.86)	12 (57.14)	10 (47.62)	2 (9.52)	-	2 (9.52)	8 (38.1)	2 (9.52)
Fresh milk	4/25 (16)	7 (33.33)	6 (28.57)	1 (4.76)	-	4 (19.05)	2 (9.52)	1 (4.76)
Raw milk	2/26 (7.69)	2 (9.52)	2 (9.52)	-	1 (4.76)	-	1 (4.76)	-
Total (%)	12/65 (18.46)	21 (100)	18 (85.71)	3 (14.29)	1 (4.76)	6 (28.57)	11 (52.38)	3 (14.29)

### Serotypes and virulence signatures of *L*. *monocytogenes* (Lm) strains

The serotypes 4b and 1/2b were detected among the isolates (S2 Fig in S1 Raw images). Serotype 1/2b (n = 18, 85.71%) was the most prevalent compared to serotype 4b (n = 3, 14.29%). The virulence determinants including *prf*A, *plc*A, *plc*B, *inl*A, *inl*C, *hly*, *mpl*, *act*A, *inl*J and *inl*B were detected in all Lm isolates recovered from the samples (S3a-S3h Fig in S1 Raw images). The Genotypic virulence profile of the listeriosis agent is presented in Table 1.

### Evaluation of the biofilm-forming potential of *L*. *monocytogenes* (Lm) isolates

The ability of Lm isolates from milk and milk products were assessed for biofilm formation. Lm isolates showed varying biofilm-forming strength. The analysis revealed that (n = 20, 95.24%) of the isolates possess biofilm-forming potentials. Six of the isolates (n = 6, 28.57%) are weak, (n = 11, 52.38%) medium and (n = 3, 14.29%) strong biofilm formers. All isolates from cheese and pasteurized milk showed varying degrees of biofilm-forming potentials (Table 1). The mean and standard deviation of the data generated from the microtiter plate biofilm production assay of each isolate was presented in (Table 2).

**Table 2:** Classification of biofilm formation of *L*. *monocytogenes* isolates recovered from milk and milk product.

Isolates no	Cheese (mean standard deviation)	Classification	Fresh milk (mean standard deviation)	Classification	Raw milk (mean standard deviation)	Classification
1	1.224 ± 09	Medium	1.7 ± 28	Strong	1.39 ± 19	Medium
2	1.023 ± 18	Weak	1.094 ± 10	Weak	0.428 ± 02	Negative
3	1.241 ± 31	Medium	1.24 ±17	Medium	-	-
4	1.38 ± 23	Medium	0.89 ± 08	Weak	-	-
5	0.956 ± 12	Weak	1.49 ± 14	Medium	-	-
6	1.4 ± 44	Medium	1.01 ± 0.9	Weak	-	-
7	1.78 ± 94	Strong	0.97 ± 13	Weak	-	-
8	1.34 ± 16	Medium	-	-	-	-
9	1.32 ± 02	Medium	-	-	-	-
10	1.228 ± 09	Medium	-	-	-	-
11	1.221 ± 05	Medium	-	-	-	-
12	1.634 ± 28	Strong	-	-	-	-

*There is a significant difference (p ≤ 0.01) between the means for raw milk compared with cheese and fresh milk.

### Antibiotic susceptibility and resistance of *L*. *monocytogenes* (Lm) isolates

**Fig 2:**
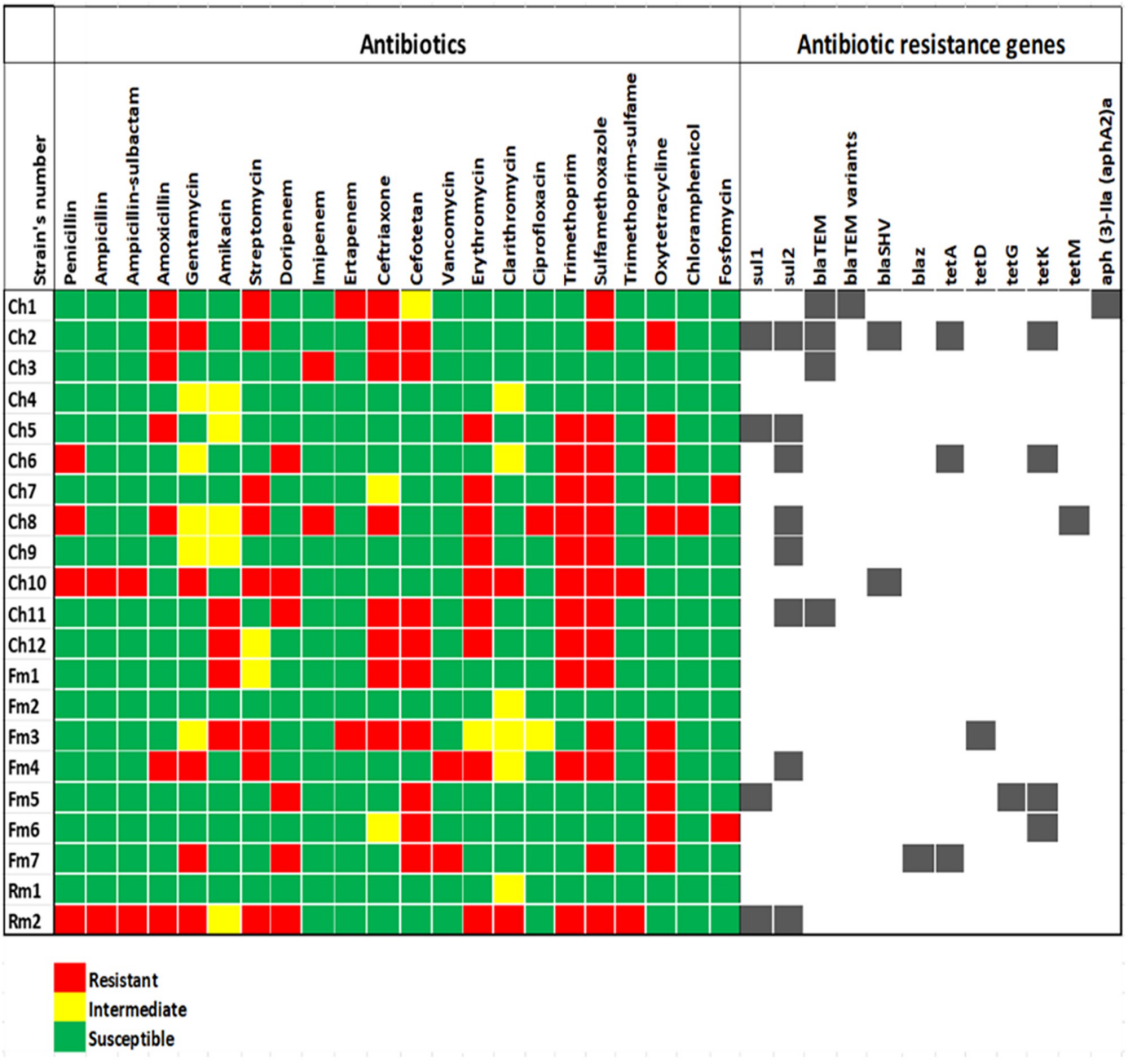
The phenotypic and genotypic antibiogram profile of *L*. *monocytogenes* strains recovered from dairy samples. The colour codes indicate resistant, intermediate, and susceptible phenotypes to the antibiotics. The code (Ch) denotes Lm strains recovered from cheese, (Fm) for fresh milk and (Rm) for raw milk. Strains Ch2, Ch8, Ch10, Fm3, Fm4, Fm6, Fm7 and Rm2 showed resistance against two or more classes (multidrug resistance, MDR) of antibiotics. The black points indicate ARGs detected in each strain including tetracycline resistance protein (*tet*A, *tet*D, *tet*G, *tet*K and *tet*M); sulphonamide-resistant dihydropteroate synthase 1 and 2 (*sul*1 and *sul*2); Temoneira β-lactamase (*bla*_TEM_) ESBL (including TEM-1 and TEM-2 variants); penicillin-hydrolyzing class A β-lactamase PC1 (*bla*_Z_); sulfhydryl variable class A broad-spectrum β-lactamase (*bla*SHV-1) and aminoglycoside β-phosphotransferase *(aph (3)-IIa (aphA2)*^*a*^.

The susceptibility of Lm to 22 panels of antibiotics commonly used to alleviate microbial infections was assessed. The isolates exhibited varying susceptibility (> 50%) to all antibiotics except trimethoprim (47.61%) and sulfamethoxazole (28.57%) whereas, high resistance against sulfamethoxazole (71.43%), trimethoprim (52.86%); erythromycin, cefotetan and oxytetracycline (42.86% respectively) was observed. On the other hand, intermediate resistance (> 20) among the macrolides, aminoglycosides, cephalosporins and the fluoroquinolones antibiotics (S3b Table in S1 File) was observed including clarithromycin (28.57%), gentamicin and amikacin (23.81% respectively). The heatmap in Fig 2. gave a descriptive representation of the typical phenotypic antibiotic susceptibility pattern of each of the Lm isolates. This pattern observed could reflect the genetic attributes as it revealed the effectiveness of the antibiotics towards each isolate. The prevalence of resistant Lm isolates in the samples ranged from 1 to 10. High prevalence of vancomycin, ciprofloxacin, trimethoprim, ceftriaxone, and trimethoprim-sulfamethoxazole resistant isolates were observed in cheese and pasteurized milk (Table 3). There is a correlation (P < 0.01) in the distribution of resistant Lm in milk and milk products.

**Table 3:** Distribution of antibiotic-resistant isolates *L*. *monocytogenes* (Lm) isolates in dairy samples.

Samples	No	P	AMP	SAM	AML	CN	AK	S	DOR	IPM	ETP	CRO	CTT	VA	E	CLA	CIP	W	RL	TS	OT	C	FOS
Cheese	12	1	1	5	2	2	5	3	2	1	6	4	0	7	1	1	8	10	1	4	1	1	1
Fresh milk	7	0	0	1	2	2	2	2	0	1	2	5	2	1	0	0	2	4	0	5	0	1	0
Fresh milk	2	1	1	1	1	0	1	1	0	0	0	0	0	1	1	0	1	1	1	0	0	0	1

Penicillin G (P), ampicillin (AMP), ampicillin-sulbactam (SAM), amoxicillin (AML), gentamicin (CN), amikacin (AK), streptomycin (S), doripenem (DOR), ertapenem (ETP), imipenem (IPM), ceftriaxone (CRO), cefotetan (CTT), vancomycin (VA), erythromycin (E), clarithromycin (CLA), ciprofloxacin (CIP), trimethoprim (W), sulfamethoxazole (RL), trimethoprim-sulfamethoxazole (TS), oxytetracycline (OT), chloramphenicol (C), fosfomycin (FOS).

**There is a correlation (P < 0.01) in the distribution of phenotypically resistant Lm isolates in milk and milk products.

### Evaluation of [(M)ARPs and (M)ARI] of *L*. *monocytogenes* (Lm) isolates

The patterns of (M)ARPs and (M)ARI is represented in Table 4. Eighteen resistance patterns unique per isolate against the antibiotics tested were observed ranging from 3 to 12 antibiotics among the isolates. Three isolates (n = 3, 14.29%) recovered from each of the samples were not resistant against any of the antibiotics, 18 (85.71%) exhibited multiple antibiotic resistance while 8 (38.10%) showed multidrug-resistance phenotypes against the antibiotics. The ARI of all the samples is greater than the permissible (0.2) Krumperman threshold (0.26, 0.21 and 0.27) for cheese, fresh milk, and raw milk, respectively.

**Table 4:** Antibiotic resistance phenotypes and patterns of *L*. *monocytogenes* isolates.

**Sample**	**Isolates no**	**Serotypes**	**Resistance patterns**	**No of antibiotics**	**MARI**
Cheese					
A	1	2b	AML-S-ETP-CRO-RL	5	0.23
	2	2b	AML-CN-S-CRO-CTT-RL-OT	7	0.32
	3	2b	AML-IPM-CRO-CTT	4	0.18
B	4	2b	-	-	-
	5	2b	AML-E-W-RL-OT	5	0.23
C	6	2b	P-DOR-W-RL-OT	5	0.23
D	7	4b	S-E-W-RL-FOS	5	0.23
	8	2b	P-AML-S-IPM-CRO-E-CIP-W-RL-OT-C	11	0.50
E	9	2b	E-W-RL	3	0.14
	10	2b	P-AMP-SAM-CN-S-DOR-E-CLA-W-RL-TS	11	0.50
F	11	2b	AK-DOR-CRO-CTT-E-W-RL	7	0.32
	12	4b	AK-CRO-CTT-E-W-RL	6	0.27
	ARI		69 / 22 × 12 = 0.26	69	-
Fresh milk					
A	1	2b	AK-CRO-CTT-W-RL	5	0.23
	2	2b	-	-	-
B	3	2b	AK-S-ETP-CRO-CTT-RL-OT	7	0.32
	4	2b	AML-CN-S-VA-E-W-RL-OT	8	0.36
C	5	2b	DOR-CTT-OT	3	0.14
	6	4b	CTT-OT-FOS	3	0.14
D	7	2b	CN-DOR-CTT-VA-RL-OT	6	0.27
	ARI		32 / 22 × 7 = 0.21	32	-
Raw milk					
A	1	2b	-	-	-
B	2	2b	P-AMP-SAM-AML-CN-S-DOR-E-CLA-W-RL-TS	12	0.55
	ARI		12 / 22 × 2 = 0.27		
**Distribution of multiple antibiotic resistance of *L*. *monocytogenes* in milk and milk product samples**						
**S/N**	**MARPs patterns**	**No of antibiotics**	**Cheese**	**Fresh milk**	**Raw milk**
1	E-W-RL	3	1		
2	CTT-OT-FOS	3		1	
3	DOR-CTT-OT	3		1	
4	AML-IPM-CRO-CTT	4	1		
5	S-E-W-RL-FOS	5	1		
6	P-DOR-W-RL-OT	5	1		
7	AML-E-W-RL-OT	5	1		
8	AK-CRO-CTT-W-RL	5		1	
9	AML-S-ETP-CRO-RL	5	1		
10	AK-CRO-CTT-E-W-RL	6	1		
11	CN-DOR-CTT-VA-RL-OT	6		1	
12	AK-DOR-CRO-CTT-E-W-RL	7	1		
13	AK-S-ETP-CRO-CTT-RL-OT	7		1	
14	AML-CN-S-CRO-CTT-RL-OT	7	1		
15	AML-CN-S-VA-E-W-RL-OT	8		1	
16	P-AML-S-IPM-CRO-E-CIP-W-RL-OT-C	11	1		
17	P-AMP-SAM-CN-S-DOR-E-CLA-W-RL-TS	11	1		
18	P-AMP-SAM-AML-CN-S-DOR-E-CLA-W-RL-TS	12			1

### Evaluation of the antimicrobial resistance signatures of *L*. *monocytogenes* (Lm) isolates

Twelve (12) ARGs were detected among the isolates out of 44 genes screened. The β-lactamase resistance genes were screened among 14 isolates that expressed phenotypic resistance to β-lactam antibiotics and (n = 4, 35.71%) were positive for the genes. Four antibiotic-resistant isolates recovered from cheese (n = 4, 28.57%) were positive for *bla*_TEM_ (n = 4, 28.57%), (n = 2, 14.29%) were positive for *bla*_SHV_ and *bla*_TEM_ variants (TEM-1 and TEM-2) ARGs whereas *bla*_*Z*_ was detected in (n = 1, 7.14%) strain recovered from fresh milk. No β-lactam resistance gene was detected among resistant isolates from raw milk. Genes encoding tetracycline including the *tet*A (n = 3, 33.33%), *tet*D, *tet*G and *tet*M (n = 1, 11.11% respectively) were detected among tetracycline-resistant isolates. The *tet*K (n = 4, 44.4%) was most prevalent among the Lm strains. In addition, the aminoglycoside ARGs including the *aph (3)-IIa (aphA2)*^*a*^ gene was detected only in one isolate (n = 1, 11.11%) recovered from cheese. Furthermore, the sulfonamide ARGs were detected among 9 of 13 (69.23%) strains screened for sulfonamides resistance genes. The *sul*2 gene was predominant among the isolates (n = 8, 61.54%) compared to the *sul*1 gene (n = 4, 30.77%). Eighteen genotypic resistance patterns ranging from 1–6 resistance genes were observed among strains harbouring ARGs (Table 5). The *sul*2 ARGs was observed twice in isolates from cheese and fresh milk. Generally, 15 (71.43%) of all the resistant Lm strains recovered from the samples investigated harboured resistance genes encoding resistance against various antibiotics. The statistical analysis revealed that the relationship between the distribution of resistance genes *tet*A and *sul*2 genes are statistically significant (P < 0.01).

**Table 5:** Antibiotic resistance genotype profiles of *L*. *monocytogenes* recovered from milk and milk products.

**Strain no**	**Antibiotic resistance genes**			**No of resistance genes**	
Cheese					
1	*bla*_TEM_*-aph (3)-IIa (aphA2)*^*a*^-TEM			3	
2	*tet*A-*tetK*-*sul1*-*sul2*-*bla*_TEM_-SHV			6	
3	*bla*_TEM_			1	
4	-			-	
5	*sul*1-*sul*2			2	
6	*tet*A-*tet*K-*sul*2			3	
7	-			-	
8	*tet*M-*sul*2			2	
9	*sul*2			1	
10	SHV			1	
11	*sul*2-*bla*_TEM_			2	
12				-	
Fresh milk					
1	-			-	
2	-			-	
3	*tet*D			1	
4	*sul*2			1	
5	*tet*K-*tet*G-*sul*1			3	
6	*tet*K			1	
7	*tet*A-*bla*_Z_			2	
Raw milk					
1	-			-	
2	*sul*1-*sul*2			2	
**Genotypic resistance patterns of *L*. *monocytogenes* isolates**					
**S/N**	**Genotypic resistance patterns**	**No of resistance genes**	**Cheese**	**Fresh milk**	**Raw milk**
1	*tet*D	1		1	
2	*tet*K	1		1	
3	*sul*1	1			
4	*sul*2	1	1	1	
5	SHV	1	1		
6	*bla*_TEM_	1	1		
7	*sul*1-*sul*2	2	1		1
8	*tet*A-*bla*_Z_	2		1	
9	*tet*A-*str*A	2			
10	*tet*A-*sul*1	2			
11	*tet*M-*sul*2	2	1		
12	*sul*2-*bla*_TEM_	2	1		
13	*bla*_TEM_-SHV	2			
14	*bla*_TEM_-TEM	2			
15	*tet*K-*tet*G-*sul*1	3		1	
16	*tet*A-*tet*K-*sul*2	3	1		
17	*tet*A-*tet*K-*sul*1-*sul*2-*bla*_TEM_-SHV	6	1		
18	*bla*_TEM_*-aph (3)-IIa (aphA2)*^a^-TEM	3	1		

## Discussion

The U.S recommended standard (zero tolerance) of Lm in ready-to-eat food was adopted to assess the safety of milk and milk products investigated in this study. Going by this standard, a reasonable proportion (12/65) of the samples failed the required zero presence of Lm in the samples. Such dairy products could pose a health threat to the consumer. The prevalence of Lm in all the dairy samples investigated in our study was 18.46%. A previous study elsewhere reported a similar prevalence (18.6%) of *Listeria* spp. from various milk samples in Tehran Province, Iran [41]. Cheese (42.86%) recorded the highest prevalence among the dairy samples investigated while the prevalence in pasteurized milk and raw milk was 16% and 7.69% respectively. However, there is a paucity of information on the prevalence of Lm in the Republic of South Africa. A lower prevalence of Lm in cheese (7.4%), milk (3.0%) and zero prevalence in clotted cream samples was reported elsewhere [20]. Braga et al. observed a 10% prevalence of Lm in cheese samples in Uruguay [42]. Also, Soni et al. reported a prevalence of 5.8% in milk samples and zero prevalence of Lm in cheese, butter and ice cream [43]. Furthermore, a prevalence of 1.09% was reported in raw milk in India [44]. The high prevalence of Lm in pasteurized milk and cheese observed in our study could explain the ability of the pathogen to thrive and replicate at refrigeration temperatures during preservation. In addition, pasteurization may not have eliminated Lm in the milk during processing. Although, there are chances of post pasteurization contamination occurring in the product. Notwithstanding, studies have shown that application of pasteurization in dairy production has reduced infant and childhood mortality significantly since the late 19^th^ century [45, 46].

All isolates from samples were classified as serotype 1/2b (85.71%) and serotype 4b (14.29%). The distribution of these serotypes in milk and milk products is predictable because serotype 1/2b are frequently isolated in food samples [47]. Muraoka et al. reported a similar trend of serotype 1/2a (89.1%) and serotype 4b (10.9%) from bulk milk in the Pacific Northwest was observed [48]. Also, [43] reported 57.1% of serotype 1/2b and 42.9% of serotype 1/2a among isolates recovered from milk samples in India. Furthermore, Braga et al. observed 45. 83% of serotype 1/2b and 41.67% of serotype 4b as the most frequently observed strains isolated from cheese in Uruguay [42]. However, Shama et al. documented a much higher prevalence of serotype 4b compared to 1/2a from milk samples in India [44]. Serotype 1/2a and 4b are important epidemiological strains that cause listeriosis. Serotype 4b have been reported in several listeriosis outbreaks traced to the product of dairy including the Mexican-style cheese [16, 49], Vacherin Mont d’Or cheese [50], blue mould cheese [51], soft cheese [52], in some countries. Listeriosis outbreak involving serotype 1/2b from cheese has also been reported [53]. Also, serotype 1/2b has been found in cases of bovine mastitis [54]. The presence of these serotypes in dairy samples suggest that they could constitute a potential health threat to consumers.

Lm pathogenicity is usually associated with the virulent determinants it harbours. The virulence genes including *prf*A, *plc*A, *plc*B, *inl*A, *inl*C, *hly mpl*, *inl*B, *inl*J, and *act*A were detected in all Lm isolates recovered from milk and milk products. A similar trend was observed in a previous study where *inl*A, *inl*C, *inl*J, was detected in all isolates and the *plc*A, *prf*A and *act*A were detected among the majority of isolates from milk samples at Varanasi, India [43]. Also, [55] reported the prevalence of *act*A, *hly* and *inl*B genes (100%), (92.59%) and (81.5%) respectively in isolates from milk and other samples. The LIPI-1 virulence gene cluster plays a major role in the intracellular life cycle of the pathogen and the major steps of cellular infections [56]. In addition, cellular adhesion, and internalization within the epithelial cell of the host are coordinated by the internalin genes. Thus, the presence of LIPI-1 and the internalin virulence determinants highlights the potential virulence status of the isolates and the potential health threat associated with contaminated milk products.

Most of the Lm isolates (95.24%) demonstrated biofilm-forming potentials. This characteristic enables Lm to colonize food processing facilities for a long time, especially in regions with difficult or limited access to cleaning agents and disinfectants [10]. The presence of the LIPI-1 and *hly* virulence genes was identified to influence the effective biofilm-forming potentials in Lm [57]. This confers the ability to withstand ineffective disinfection during cleaning activities and oftentimes leads to the continuous presence of Lm in the final product. The prevalence of MDR strains observed in our study could be linked with the potential biofilm-forming ability of the isolates as it is established that biofilm-forming bacteria have a higher tolerance against disinfectants and clinical antimicrobials probably due to frequent exposure to sanitisers below recommended concentrations by manufacturers [58]. The expression of biofilm-forming potentials of Lm isolates from cheese and pasteurized milk could suggest contamination emanating from the food processing plant/environment. As such, this could in turn compromise the safety of the products for consumption.

The antibiogram profiles of Lm was assessed and variable susceptibility patterns were observed. Susceptibility patterns of Lm to antibiotics is geographically biased based on differences/non-consistent susceptibility patterns being observed across regions. In this report, high resistance against sulfamethoxazole (71.43%), trimethoprim (52.86%), erythromycin, cefotetan and oxytetracycline (42.86% respectively) were observed. Previous studies also described a high resistance against erythromycin and oxytetracycline in dairy isolates in South Africa and Turkey [18, 20]. Unfortunately, erythromycin, a drug of choice recommended for treating listeriosis in pregnant women might no longer be effective if administered for treatment in pregnant women in the catchment area. Resistance against the aminoglycosides, cephalosporins and the tetracyclines antibiotics were more frequently observed among Lm strains. A similar narrative was observed in another study in Egypt [55]. The intermediate resistance observed for clarithromycin, gentamicin and amikacin could suggest that Lm is gradually developing resistance against the antibiotics. A prevalence (38.10%) of MDR phenotypes against the antibiotics tested was observed among Lm strains recovered from dairy samples in our study. Similarly, 36.71% prevalence of MDR Lm isolates recovered from dairy products was seen in Turkey [19]. The highest resistance pattern (resistance against 12 antibiotics) was observed in isolates recovered from fresh milk. This strain exhibiting multiple antibiotic resistance might have originated from dairy animals and such resistance might be acquired through frequent exposures to antibiotics used in animal husbandry. The MARI indices strongly suggest the level of resistance of each isolate and varied among the isolates as it reflects the number of phenotypic resistances recorded with respect to antimicrobial agents tested. Isolates with high MARI scores suggest that they were resistant against many antibiotics vice versa. Consequently, MDR resistant bacteria is usually associated with difficulty in treating infections they caused as it could translate to higher hospitalization costs and extended duration of antibiotic administration [59]. The MDR strains are most often abundant in the environment and could gain access to food processing plants via factory personnel or incoming raw materials [18]. Several factors have been identified influencing antimicrobial resistance in Lm, especially in the food sector. In addition, exposure to food processing stress could facilitate the development of resistance to clinically relevant antibiotics [23].

Antimicrobial resistance genes (ARGs), encoded on mobile genetic elements are fundamental to the acquisition of antibiotic resistance in Lm. Acquisition of these genetic elements is mediated by horizontal gene transfer based on evidence of homologous recombination, presence of plasmid, conjugative transposons, and prophages [23]. ARGs encoding resistance against various classes of antibiotics including sulfonamides, β-lactams, tetracycline, and aminoglycoside were detected among Lm isolates recovered from dairy products in our study. Previous studies have reported the presence of various ARGs detected in Lm including the *tet*A, *str*A, *sul*I, *pen*A, and *flo*R among dairy isolates [60]. Also, the *tet*S, *tet*M, the *aad*A gene encoding streptomycin adenylyltransferase, the *cat* gene that encodes acetyltransferase responsible for the catalysis of acetyl-*S*-coenzyme A (CoA)-dependent acetylation of chloramphenicol at the 3-hydroxyl group was reported among clinical isolates of Lm [61]. Also, a report of the *erm*(B) gene encoding a 23S rRNA methyltransferase that modifies the antibiotic binding site of macrolide-lincosamide-streptogramin B (MLS_B_) among Lm isolates from humans was documented [61]. Furthermore, a report of plasmid-mediated resistance (genes in pIP811) conferring resistance against streptomycin, erythromycin and chloramphenicol among clinical isolates was earlier reported [62]. Beyond this, the possibility for transfer of ARGs (erythromycin and tetracycline ARGs) from lactic acid bacteria (LAB) to Lm within fermenting milk was identified [63]. In addition, the conjugative transfer of the Tn*6198* ARG encoding resistance against trimethoprim between *Enterococcus faecalis* and Lm in surface inoculated cheese and smoked salmon has been documented [64]. Furthermore, a report identified that sublethal stress including increased osmotic stress, low temperature and reduced pH relevant to the preservation of food may influence conjugative plasmid exchange of ARGs [23]. Bae et al. also demonstrated that efflux pump activity contributes to increased antimicrobial resistance, especially in MDR Lm [65]. The phenotypic resistance among MDR Lm that do not harbour ARGs that encode such resistance could be attributed to active efflux pump activity.

Our study revealed the occurrence, virulence determinants and genetic characteristics and the antibiogram signatures of Lm recovered from milk and dairy products in the ECPSA. However, a major limitation of the study is the small sample group tested as well as the inability to differentiate between Lm strains isolated from the same sample to their respective clones as this may lead to overestimation of the same clone. We recommend that future studies should consider a more representative sample size to allow for a more reliable conclusion.

### Control of antimicrobial resistance and food safety—A One Health perspective

Rapid and intense alteration of the socio-ecology is impacting more severely on humans and animal health. The emerging threat pose by the increasing rates of antimicrobial resistance among infectious bacteria is worrisome and demand urgent attention. The development of resistance against medically important antibiotics is largely dependent on the indiscriminate use of antibiotics in humans and animal husbandry. Most of the antibiotics are usually excreted unchanged into the environment thereby increasing concerns about the potential residual impact of these agents in the environment [39, 66] and also in foodstuffs such as meat, egg [67] and milk as a result of treatment for mastitis in cattle [68]. Also, frequent exposure of microorganisms to antibiotic residues (concentration below the lethal dose) in the environment could facilitate antibiotic resistance. This threatens not only humans, (including economies and food supplies) but, also the microbial communities and the essentially critical biodiversity that bear the living infrastructure of our world. The residual impact of antibiotics could result in extreme antibiotic resistance, immunopathological effects, mutagenicity, allergy, hepatotoxicity, nephropathy (gentamicin), bone marrow toxicity (chloramphenicol), carcinogenicity (furazolidone, oxytetracycline, sulfamethazine), reproductive disorder, transfer of antibiotic-resistant bacteria to humans [67, 69] ecological toxicity, high drug failures, and even ARGs selection [70, 71]. As such, a multi-sectorial innovative and collaborative approach (One Health) that came to the limelight in 2004 held at the Rockefeller University, Manhattan [72] and the updated Berlin principles [73] becomes imperative to addressing the global health challenge. The fifth and eighth of the Berlin principles are especially relevant to the control of antimicrobial resistance in microorganisms including Lm in the food web. These are: that adaptive, holistic, and progressive approaches to detect, prevent, monitor, control and alleviate emerging diseases (communicable and non-communicable diseases) that include the composite interconnections among species, ecosystem and human society while accounting fully for harmful economic drivers and perverse subsidies; Increased capacity for cross-sectorial and trans-disciplinary health surveillance and clear, timely information sharing to improve coordination of responses among governments and NGOs, health, academia and other institutions, industries and stakeholders [73]. In this background, food safety, welfare and public health would only be achievable in the coming decades only on principles that operate successfully on the One Health approach [74].

Quite a number of zoonoses of public health significance are foodborne, although, some foodborne pathogens may be non-zoonotic [74]. As such, allocating food safety resources where the focus is geared mostly towards contributing to One Health benefit is key. The food safety policy to control Lm in milk and milk products should take source attributes including health and welfare of animals, contamination/safety of equipment at processing facilities to ensure best practices for producers and improve milk quality and production. Also, post-processing contamination from the environment and cross-contamination from processing plants are major concerns for Lm in the dairy sector. Although, Lm could be inactivated in food by thermal treatments (pasteurization) during processing [75]. Ensuring animal health, especially in dairy animals as such that lactating cows were identified to shed Lm in milk for a long time. Also, proper milking procedures, equipment maintenance, dry cow therapy, biosecurity, management of clinical mastitis, good record keeping and consumer preferences are required [68, 76, 77]. In like direction, control of Lm contamination in feed/silage, and use of high water quality are critical to maintaining healthy dairy animals and safeguarding dairy animals/products from disease and chemical adulterants [68]. The control of Lm in the dairy sector is required at all stages through an integrated approach to preventing the contamination and multiplication of the Lm in the final product. Proper implementation and enforcing the correct functioning of the HACCP (Hazard Analysis and Critical Control Point) principles and the GMPs (Good Manufacturing Practices) tailored to dairy producers’ specific needs by government agencies in food [78, 79] and dairy production are primary to achieving safe food for the consumers, especially in developing countries where regulation enforcement may be lacking. Furthermore, hygiene regulations (Good Hygienic Practices—GHP) and surveillance for the presence of Lm in dairy facilities and processing areas are achievable through good equipment design. Monitoring dairy products (product testing at intervals along the production and distribution line) to conform with the zero-tolerance of Lm in ready-to-eat food is the right step towards achieving food safety [79, 80]. Furthermore, limiting and preventing the rapid spread of antimicrobial resistance and ARGs by adopting organic farming, strict policies against the magnanimous usage of antibiotics in animal husbandry to avoid antibiotic residue in dairy products is critical to achieving the safety of milk and milk products for human consumption.

## Conclusion

Milk constitutes a significant proportion of the most widely consumed animal protein; thus, the economic and dietary importance cannot overemphasize. The presence of antimicrobial-resistant Lm and ARGs in milk products is a potential threat to human health. This, in part, is a challenge to ensuring the availability of safe and wholesome food for the teeming human population without escalating the impact of food production and consumption on the environmental footprint. Adopting the One Health approach to achieving food safety, public health and welfare would largely depend on the strength of collaboration among researchers, industries, stakeholders, national agencies, and political officeholders. Successes over future challenges would be possible with close One Health collaboration across boards.

## Supporting information

S1 File SI tables—Contains all supporting tables. (DOCX)

S1 Raw images SI raw gel images—Contains all raw gel images. (PDF)

S1 Dataset Minimal data set—Contains raw data set. (CSV)
