# Supplementary material for: Assessment of multidrug-resistant Listeria monocytogenes in milk and milk product and One Health perspective
Source: PLoS One. 2022 Jul 6;17(7):e0270993. doi: 10.1371/journal.pone.0270993 (PMC9258876; doi:10.1371/journal.pone.0270993)
Supplement: S1 File — (DOCX) [file pone.0270993.s001.docx]

**S1 Table. Description of sampling locations**

| **Municipality Districts** | **Location code** | **Town/city** | **Description** | **Geographical coordinates** |
| --- | --- | --- | --- | --- |
| Amathole | S1 | East London | East London is a city on the Indian Ocean situated 1000 kilometres (km) from Cape Town between Nahoon River and the Buffalo River to the South inhabited by the Xhosa-speaking people. It is known for its Nahoon and Cove Rock beaches | 33.0292° S, 27.8546° E |
|  | S2 | Mdantsane | An urban township and the second largest township in Eastern Cape situated 15 km away from East London | 32.9460° S, 27.7256° E |
|  | S3 | King William’s Town | Is a city of about 35,000 inhabitants situated along the banks of the Buffalo River about 60 km northeast of the Indian Ocean | 32.8938° S, 27.4204° E |
|  | S6 | Butterworth | A town situated on the N2 national highway accommodating about 45,900 inhabitants and populated by the AmaXhosa, KhoiKhoi and San people | 32.3323° S, 28.1446° E |
|  | S8 | Alice | A small town named after Princess Alice; daughter of the British Queen Victoria adjacent to the Tyhume River. The University of Fort Hare is situated in the community | 32.7901° S, 26.8330° E |
|  | S9 | Adelaide | A rural town and are situated near the Great Winterberg mountain range | 32.7071° S, 26.2952° E |
|  | S10 | Keiskammahoek | A town part of the Ciskei Bantustan situated 40 km to the west of King William’s Town. It is surrounded by a number of villages in the peri-urban settlements situated near the Keiskamma River | 32.6849° S, 27.1324° E |
|  | S11 | Bedford | Is a village situated at the junction of the R360 from Grahamstown and the R63 between Adelaide and Somerset East | 32.6879° S, 26.1064° E |
|  | S13 | Cathcart | It was named after Sir George Cathcart situated on the N6, 48 km north of Stutterheim en route to Komani | 32.2973° S, 27.1324° E |
|  | S14 | Stutterheim | Is a town with a population of 46,730 situated in the border region of the ECPSA | 32.5885° S, 27.4321° E |
| Chris Hani | S12 | Whittlesea | A semi-rural town situated in the Hewu district, 37 km south of Queenstown | 32.1760° S, 26.7909° E |
|  | S15 | Tarkastad | A Karoo semi-urban settlement situated on the banks of the Tarka River. It is located on the plain to the north of the Winterberg mountain range on the R61 between Cradock and Queenstown | 32.0079° S, 26.2718° E |
|  | S16 | Cofimvaba | A town 79 km east of Queenstown on the route to Butterworth in Thembuland | 32.0187° S, 27.5848° E |
|  | S17 | Cradock | A town in the upper valley of the Great Fish River, 250 km by road northeast of Port Elizabeth | 32.1810° S, 25.6510° E |
|  | S18 | Lady Frere | A small town named after the wife of Sir Henry Bartle Frere, Governor of the Cape Colony | 31.7529° S, 27.2191° E |
| Sarabartman | S4 | Grahamstown | Lies on the wooded slopes of the Suur Mountains near the source of the Kowie River. It inhabits about 140,000 people situated about 11 km northeast of Port Elizabeth and 130 km southwest of East London | 33.3106° S, 26.5256° E |
|  | S5 | Port Elizabeth | Colloquially referred to as P.E and the location for the major seaport city and the most populous city in the ECPSA. It is the seat of the Nelson Mandela Bay Metropolitan Municipality, South Africa’s second-largest metropolitan district by area size | 33.9608° S, 25.6022° E |
|  | S7 | Klipplaat | It is some 185 km north-west of Port Elizabeth and 75 km south-east of Aberdeen | 33.0249° S, 24.3401° E |
|  | 19 | Somerset East | A town in the Blue Crane Route Local Municipality founded by Lord Charles. The Blue Crane route follows the national road R63 from Pearston, via Somerset East to Cookhouse. | 32.7248° S, 25.5800° E |

Note: Description of sampling locations were represented in a map in Fig 1 and described in SI Table. Details about names of purchase locations (grocery stores and point of sale) and details about the purchased items were not provided due to ethical issues consideration. Sampling location coordinates were retrieved by “etrex-LEGENDH” GPS device.

**S2 Table. Primers for the PCR amplification of virulence genes**

| **Virulence gene** | **Primer** | **Sequence (5′-3′)** | **Amplicon size (bp)** | **Reference** |
| --- | --- | --- | --- | --- |
| *inlA* | *inlA*-F  *inlA*-R | CCTAGCAGGTCTAACCGCAC  TCGCTAATTTGGTTATGCCC | 256 | (Coroneo et al., 2016) |
| *inlB* | *inlB*-F  *inlB*-R | TGATGTTGATGGAACGGTAAT  CTCGTGGAAGTTTGTAGATGC | 272 | (Du et al., 2017) |
| *inlC* | *inlC*-F  *inlC*-F | AATTCCCACAGGACACAACC  CGGGAATGCAATTTTTCACTA | 517 | (Liu et al., 2007) |
| *inlJ* | *inlJ*-F  *inlJ*-R | TGTAACCCCGCTTACACAGTT  AGCGGCTTGGCAGTCTAATA | 238 | (Liu et al., 2007) |
| *actA* | *actA*-F  *actA*-R | CCAAGCGAGGTAAATACGGGA  GTCCGAAGCATTTACCTCTTC | 650 | (Lomonaco et al., 2012) |
| *prfA* | *prfA*-F  *prfA*-R | AACCAATGGGATCCACAAG  ATTCTGCTAACAGCTGAGC | 479 | (Jung et al., 2009) |
| *hlyA* | *hly*-F  *hly*-R | CAAACTGAAGCAAAGGATGCA  CTAATGTATTTACTGCGTTGTTA | 496 | (Jung et al., 2009) |
| *plcA* | *plcA*-F  *plcA*-R | CAGCATACTGACGAGGTGTG  GATGTCCGCTCTACCTGA | 674 | (Jung et al., 2009) |
| *plcB* | *plcB*-F  *plcB*-R | GCATGATATTGACAGCAAATTA  TGAAATACTTTGCTCCTGTT | 320 | (Jung et al., 2009) |
| *mpl* | *mpl-F*  *mpl-F* | TGTATCATCATGGTAATAGCT  TGGATCCGTAAACATATTCGT | 798 | (Jung et al., 2009) |

**S3a Table. Antibiotic breakpoints for the description of the antibiotic susceptibility testing for *L. monocytogenes***

| **Antibiotic class** | **Antibiotic agents** | **Discs’ code** | **Conc (μg)** | **Zones of inhibitions** | | |
| --- | --- | --- | --- | --- | --- | --- |
|  |  |  |  | **Susceptible** | **Intermediate** | **Resistant** |
| **β-Lactams** | Penicillin G | (P) | 10 | 13 | 11-12 | 13 |
|  | Ampicillin | (AMP) | 10 | 16 | 12-15 | 16 |
|  | Ampicillin-sulbactam | SAM | 20 | 16 | 12-15 | 16 |
|  | Amoxicillin | (AML) | 10 | 20 | 15-18 | 19 |
| **Aminoglycoside** | Gentamicin | (CN) | 10 | 17 | 15-16 | 12 |
|  | Amikacin | (AK) | 30 | 17 | 15-16 | 14 |
|  | Streptomycin | (S) | 25 | 17 | 15-16 | 14 |
| **Carbapenem** | Doripenem | DOR | 10 | 26 | 18-25 | 26 |
|  | Ertapenem | (ETP) | 10 | 26 | 18-25 | 26 |
|  | Imipenem | (IPM) | 10 | 26 | 18-25 | 26 |
| **Cephalosporin** | Ceftriaxone | (CRO) | 30 | 28 | 26-27 | 21 |
|  | Cefotetan | (CTT) | 30 | 27 | 25-26 | 24 |
| **Glycopeptide** | Vancomycin | (VA) | 30 | 17 | 15-16 | 14 |
| **Macrolides** | Erythromycin | (E) | 15 | 21 | 16-20 | 15 |
|  | Clarithromycin | CLA | 15 | 21 | 17-20 | 16 |
| **Fluoroquinolone** | Ciprofloxacin | (CIP) | 5 | 21 | 16-20 | 15 |
| **Sulfonamide** | Trimethoprim | (W) | 5 | 16 | 11-15 | 10 |
|  | Sulfamethoxazole | (RL) | 5 | 19 | 16-18 | 15 |
|  | Trimethoprim-Sulfamethoxazole | TS | 25 1.25/23.75 | 19 | 16-18 | 15 |
| **Tetracycline** | Oxytetracycline | (OT) | 30 | 28 | 25-27 | 24 |
| **Phenicol** | Chloramphenicol | (C) | 30 | 21 | 18-20 | 20 |
| **Phosphonic acid derivative** | Fosfomycin | (FOS) | 50 | 16 | 13-15 | 12 |

Antibiotic breakpoints were determined according to the Clinical and Laboratory Standards Institute and European Committee on Antimicrobial Susceptibility Testing (EUCAST) adopting the criteria set for *L. monocytogenes*. Antibiotic *Streptococcus pneumonia,* and *Staphylococcus* spp [36,37]

**S3b Table. Antibiotic susceptibility profile of *L. monocytogenes* isolates**

| **Antibiotic class** | **Antibiotic agents** | **Discs’ code** | **Conc (μg)** | **No of Susceptible isolates (%)** | **Intermediate (%)** | **No of Resistant isolates (%)** |
| --- | --- | --- | --- | --- | --- | --- |
| **β-Lactams** | Penicillin G | (P) | 10 | 17 (80.95) | **-** | 4 (19.05) |
|  | Ampicillin | (AMP) | 10 | 19 (90.48) | **-** | 2 (9.52) |
|  | Ampicillin-sulbactam | SAM | 20 | 19 (90.48) | **-** | 2 (9.52) |
|  | Amoxicillin | (AML) | 10 | 14 (66.67) | **-** | 7 (33.33) |
| **Carbapenems** | Doripenem | (DOR) | 10 | 15 (71.43) | **-** | 6 (28.57) |
|  | Ertapenem | (ETP) | 10 | 19 (90.48) | **-** | 2 (9.52) |
|  | Imipenem | (IPM) | 10 | 19 (90.48) | **-** | 2 (9.52) |
| **Macrolides** | Erythromycin | (E) | 15 | 11 (52.38) | 1 (4.76) | 9 (42.86) |
|  | Clarithromycin | (CLA) | 15 | 13 (61.91) | 6 (28.57) | 2 (9.52) |
| **Sulfonamides** | Trimethoprim | (W) | 5 | 10 (47.61) | **-** | 11 (52.38) |
|  | Sulfamethoxazole | (RL) | 5 | 6 (28.57) |  | 15 (71.43) |
|  | Trimethoprim-Sulfamethoxazole | (TS) | 1.25/23.75 | 19 (90.48) | **-** | 2 (9.52) |
| **Aminoglycosides** | Gentamicin | (CN) | 10 | 11 (52.38) | 5 (23.81) | 5 (23.81) |
|  | Amikacin | (AK) | 30 | 12 (57.14) | 5 (23.81) | 4 (19.05) |
|  | Streptomycin | (S) | 25 | 11 (52.38) | 2 (9.52) | 8 (38.10) |
| **Cephalosporin** | Ceftriaxone | (CRO) | 30 | 11 (52.38) | 2 (9.52) | 8 (38.10) |
|  | Cefotetan | (CTT) | 30 | 11 (52.38) | 1 (4.76) | 9 (42.86) |
| **Glycopeptides** | Vancomycin | (VA) | 30 | 19 (90.48) | **-** | 2 (9.52) |
| **Fluoroquinolones** | Ciprofloxacin | (CIP) | 5 | 19 (90.48) | 1 (4.76) | 1 (4.76) |
| **Tetracyclines** | Oxytetracycline | (OT) | 30 | 12 (57.14) | **-** | 9 (42.86) |
| **Phenicol** | Chloramphenicol | (C) | 30 | 20 (95.24) | **-** | 1 (4.76) |
| **Phosphonic acid derivative** | Fosfomycin | (FOS) | 50 | 19 (90.24) | **-** | 2 (9.52) |
