# Supplementary material for: Assessment of multidrug-resistant Listeria monocytogenes in milk and milk product and One Health perspective
Source: PLoS One. 2022 Jul 6;17(7):e0270993. doi: 10.1371/journal.pone.0270993 (PMC9258876; doi:10.1371/journal.pone.0270993)
Supplement: S1 Raw images — (PDF) [file pone.0270993.s002.pdf]

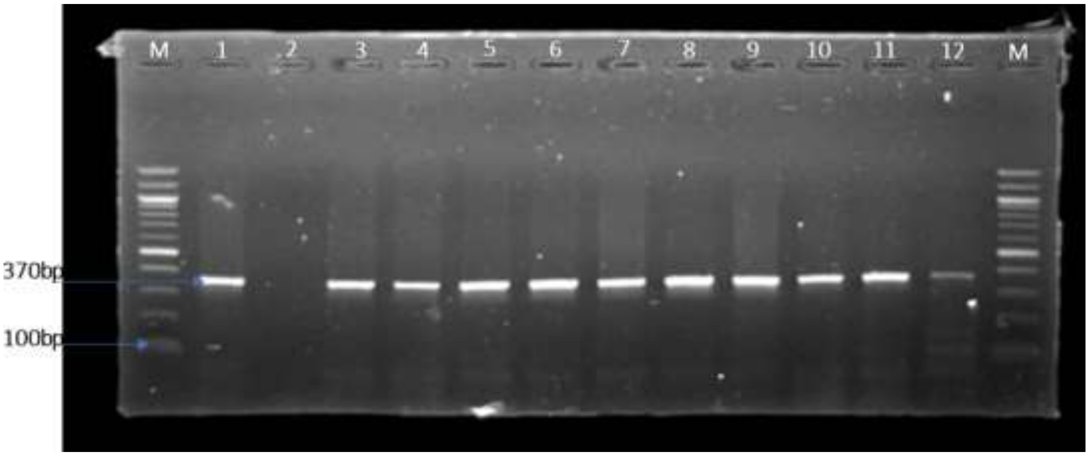

S1a Fig. Gel image of the *prs* gene (370 bp) of *Listeria* genus. Lane M: 100 bp DNA ladder, lane 1: +ve control (*Listeria. monocytogenes* ATCC 19118), lane 2: -ve control, lane 3-12 positive *Listeria* genus.

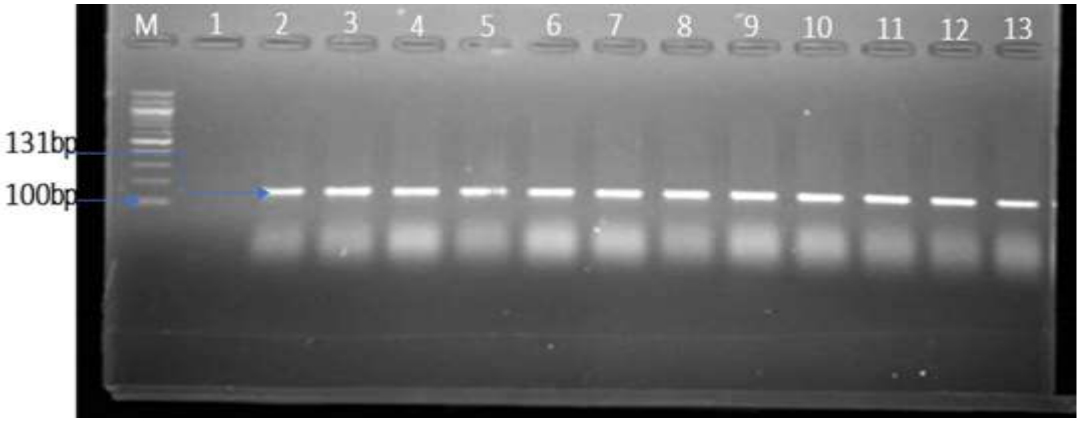

S1b Fig. Gel image of the *iap* gene (131 bp) of *L. monocytogenes*. Lane M: 100 bp DNA ladder, lane 1: -ve control, lane 2: +ve control (*L. monocytogenes* ATCC 19118), lane 3-13 positive *Listeria* genus.

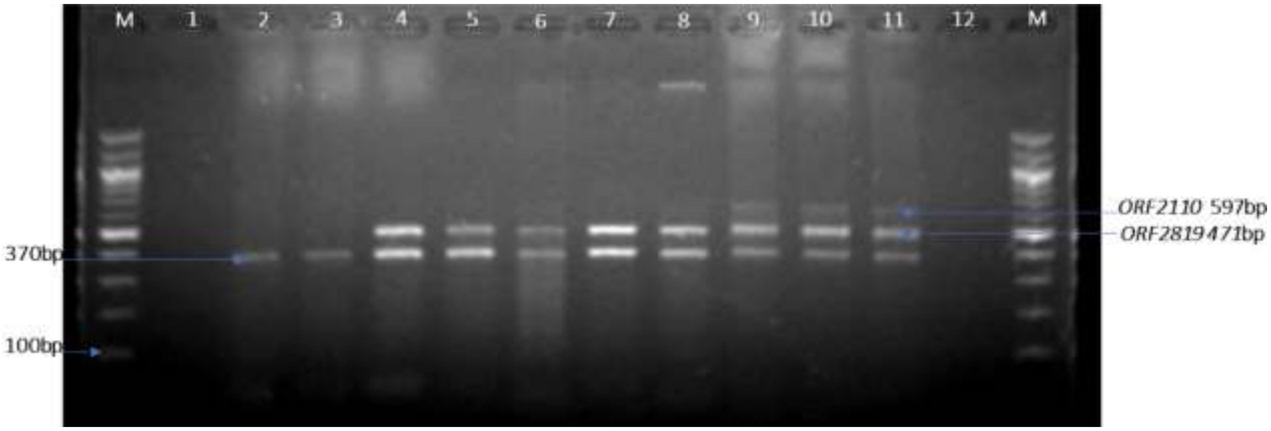

S2 Fig. Multiplex PCR of positive serotypes 4a and 1/2b strains of *L. monocytogenes.* Lane M:100 bp DNA ladder; lane 1 and 12: -ve control; lane 9 - 11: positive serotype 4b strains; lane 4 - 8: positive serotype 1/2b strains. All *Listeria monocytogenes* strains amplified the *prs* gene fragment (lane 2-11). Genes corresponding to the amplified fragment and their molecular sizes are shown on the left.

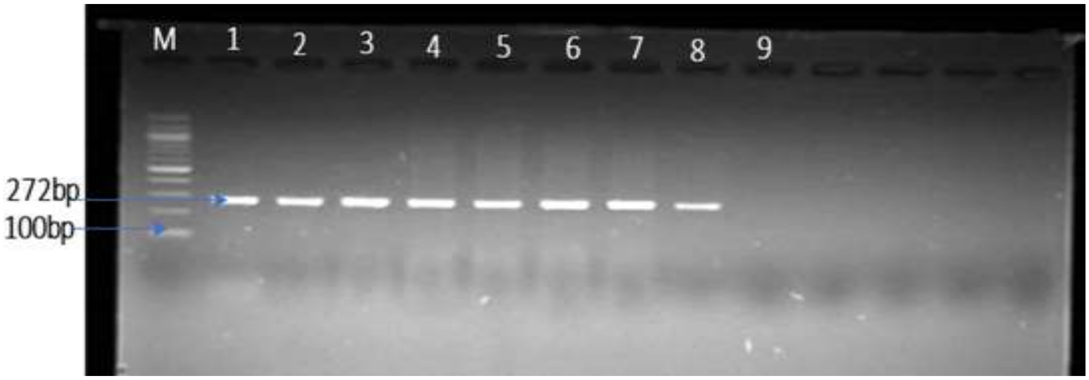

S3a Fig. Gel image of the *inl*B gene (272 bp) of *L. monocytogenes*. Lane M: 100 bp DNA ladder, lane 1-8: positive *L. monocytogenes* positive for *inl*B gene, lane 9: -ve control.

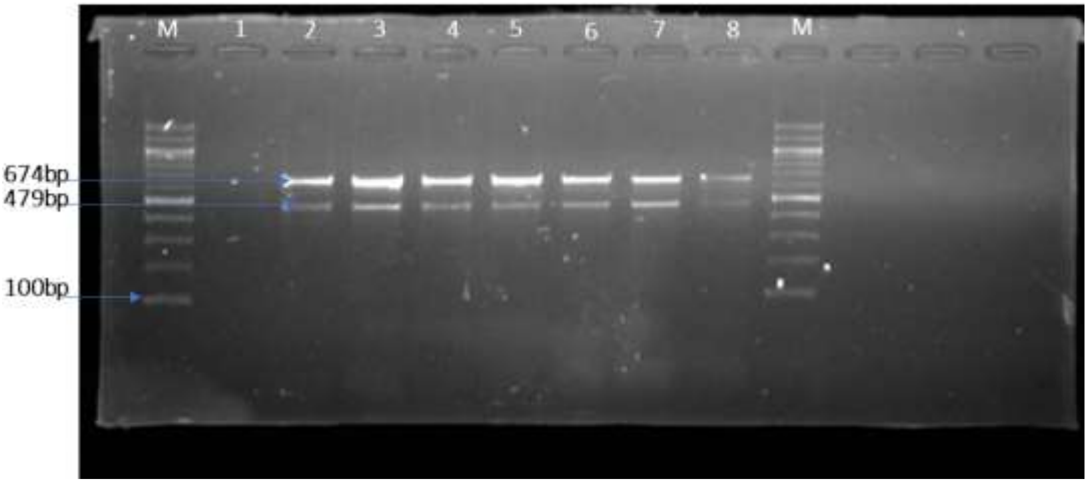

S3b Fig. Gel image of the *plc*A (674 bp) and *prf*A (479 bp) gene of *L. monocytogenes*. Lane M: 100 bp DNA ladder, lane 1: -ve control, lane 2-8 *plc*A and *prf*A genes.

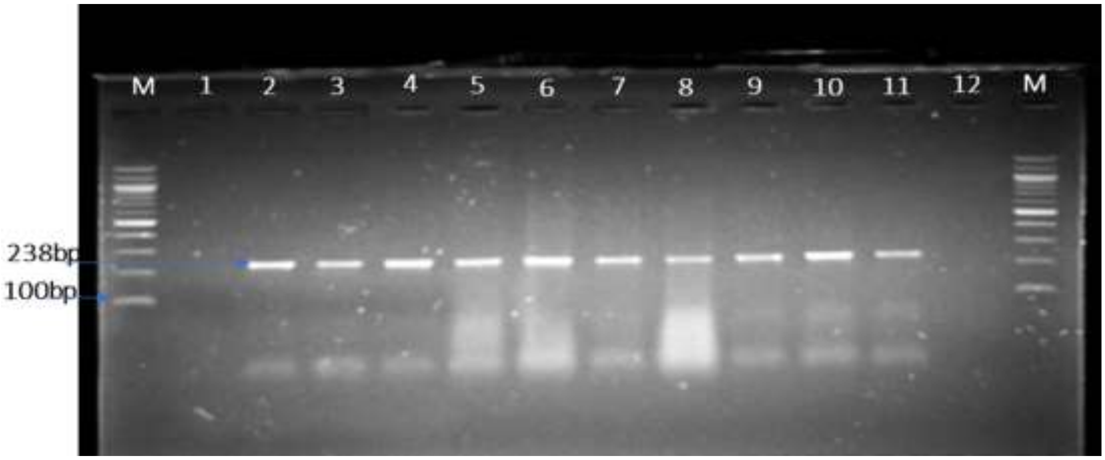

S3c Fig. Gel image of the *inl*J (238 bp) gene of *L. monocytogenes*. Lane M: 100 bp DNA ladder, lane 1: -ve control, lane 2-12 *inl*J genes.

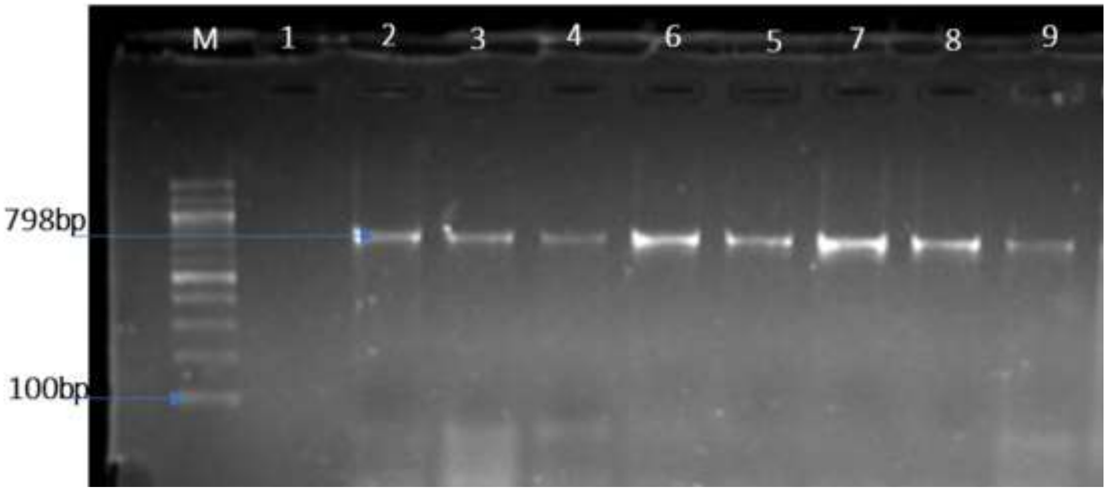

S3d Fig. Gel image of the *mpl* (798 bp) gene of *L. monocytogenes*. Lane M: 100 bp DNA ladder, lane 1: -ve control, lane 2-9 *mpl* genes.

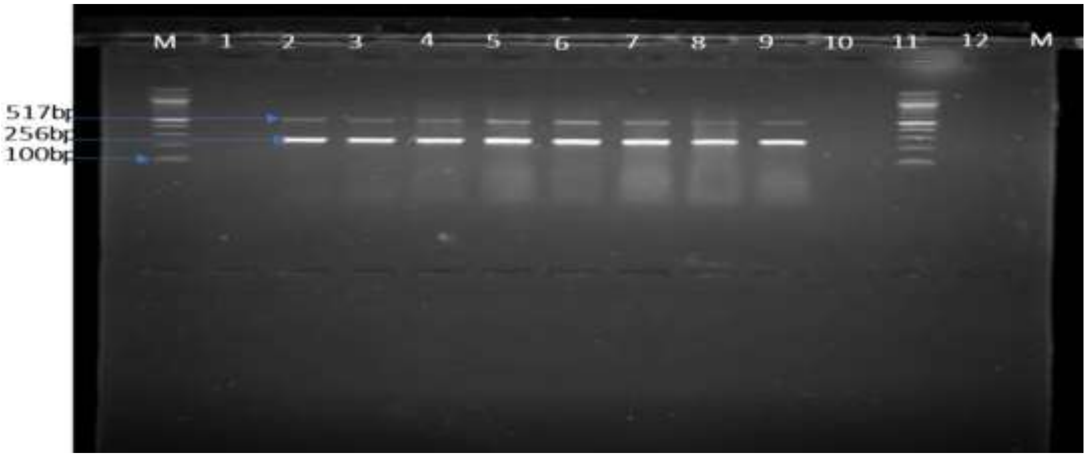

S3e Fig. Gel image of the *inlC* (517 bp) and *inl*A (256 bp) gene of *L. monocytogenes*. Lane M: 100 bp DNA ladder, lane 1 and 12: -ve control, lane 2-9 *inl*C and *inl*A genes.

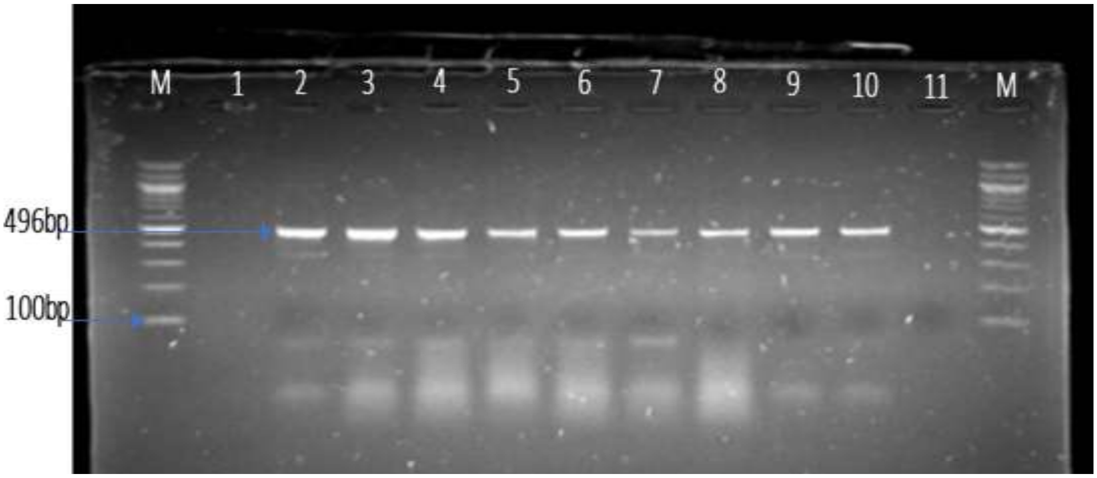

3f Fig. Gel image of the *hly*A (496 bp) gene of *L. monocytogenes*. Lane M: 100 bp DNA ladder, lane 1 and 11: -ve control, lane 2-10 *hly* gene.

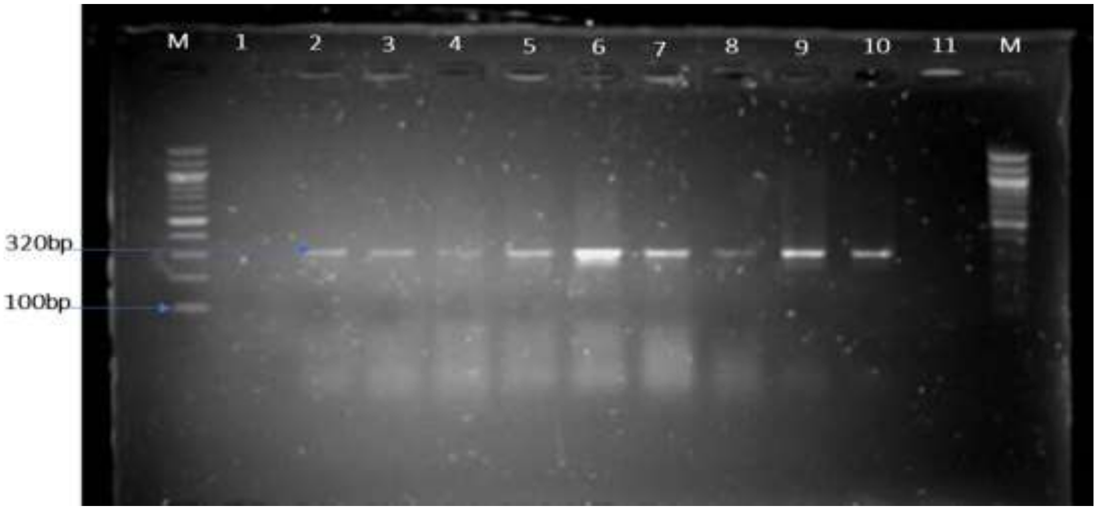

S3g Fig. Gel image of the plcB (320 bp) gene of *L. monocytogenes*. Lane M: 100 bp DNA ladder, lane 1 and 11: -ve control, lane 2-10 *plc*B gene.

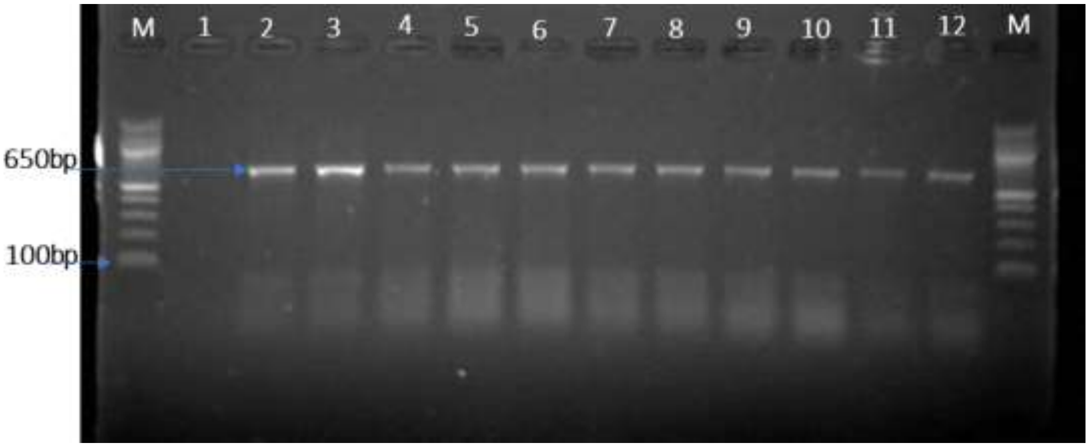

S3h Fig. Gel image of the *act*A (650 bp) gene of *L. monocytogenes*. Lane M: 100 bp DNA ladder, lane 1: -ve control, lane 2-12 *hly* gene.
